# Patient Roles and Patient Knowledge in Learning Health Systems

**DOI:** 10.1002/hpm.70019

**Published:** 2025-09-06

**Authors:** Cara Evans, Christopher Canning, Heather L. Bullock

**Affiliations:** ^1^ Waypoint Centre for Mental Health Care Penetanguishene Ontario Canada; ^2^ Department of Family and Community Medicine University of Toronto Toronto Ontario Canada; ^3^ Department of Psychiatry University of Toronto Toronto Ontario Canada; ^4^ Department of Health Research Methods Evidence and Impact McMaster University Hamilton Ontario Canada

**Keywords:** learning health systems, patient engagement, patient involvement, patient leadership

## Abstract

Learning health systems collect and analyse data on an ongoing basis to make real‐time, evidence‐informed decisions. Patient involvement is central to learning health systems. In this perspective paper, we describe implications that LHSs' distinguishing features have for patient involvement. These include the need to: build capacity for patients to engage across cycles of data collection and analysis; flesh out the role of patients with respect to collection and analysis of health system data; and create infrastructure to support involvement within learning‐intensive environments. We argue that meaningfully involving patients in LHSs requires attention to the relational and epistemological complexity of this endeavour. We conclude with six recommendations for practice, policy, and research.

## Highlights

- At present, learning health systems do not always involve patients
- Training and supports, co‐developed by patients, can support involvement
- Patient roles related to health system data need to be defined
- Meaningful involvement require attention to differing ideas about evidence

## 1 Background

The phrase “learning health system” (LHS) has been used to describe an aspirational state of ongoing, evidence‐driven improvement within health services. LHSs are characterised by learning cycles, in which data is transformed into knowledge; knowledge is translated to performance; and performance is analysed to generate new evidence [1, 2]. Patient involvement is often described as a core component of LHSs. For instance, a recent Canadian framework for LHSs visually represents “patient, caregiver, and provider co‐design” as a central gear, interlocking with other LHS functions such as data analysis and evaluation [3]. However, researchers have noted this discursive centrality is not reflected in practice. Many patients are unaware of the concept of a LHS, and few resources exist for fostering wide‐spread understanding. As a result, the uptake of this model has not consistently been driven by or inclusive of patient leadership [4].

Literature describing or defining patient roles in LHSs is scant. Broadly, current literature often describes four structures through which patients can be involved in LHSs: patient and family advisory councils [5, 6]; patient advisors embedded within governance, quality improvement, or other organizational committees [7, 8]; co‐investigator roles on research projects [9, 10]; and patient leadership [5, 11]. These structures are overlapping rather than mutually exclusive. For instance, members of project‐specific advisory councils may also serve on organisation‐wide governance structures [6, 7]. Within these structures, patients may be engaged in a range of activities such as setting priorities relating to data collection [7] and analysing data collected in research and evaluation projects [9]. In contrast to a focus on discrete roles or structures for patient involvement, Seid and colleagues (2024) describe “engagement” as a process that occurs among all actors and at all levels within a collaborative learning health system [12]. They argue that a culture of engagement enables production, dissemination, and use of knowledge across the system.

In this perspective paper, we describe the implications that LHSs' distinguishing features have for patient involvement. We argue that genuine patient involvement and leadership in LHSs can be facilitated by attending to the relational and epistemological complexity of integrating patient knowledge into health system decision‐making. Without engaging these complexities, patient involvement in LHSs may be tokenistic rather than impactful. We conclude with recommendations for future research and practice.

### 1.1 Drawing on, and Distinguishing From, Related Areas of Literature

Existing bodies of knowledge may offer frameworks, tools, and guidance to support the effective involvement and leadership of patients within LHSs. For example, recent reviews have addressed patient involvement in quality improvement [13], research [14], and health system decision‐making broadly [15]. At the same time, LHS leaders and practitioners will need to distinguish LHSs from other health system activities when discussing patient involvement.

Friedman (2022) argues that three considerations make LHSs distinct from continuous quality improvement or other similar approaches: (1) the convening of a multi‐stakeholder group that stewards the entire learning cycle; (2) the commitment to exploring evidence prior to beginning implementation; and (3) the co‐occurrence of multiple learning cycles within a supportive infrastructure [16]. With regards to the first distinguishing factor, many papers only discuss patient involvement in a single LHS activity rather than across a cycle of learning. As LHS practitioners build and define patient roles across the full scope of learning activity, literature on patient involvement in other domains may offer important insights. For instance, role clarity has been found to be critical for patients who are involved as partners in research [15]. This suggests that patient roles will need to be defined within each learning stage. In one example of this, a document produced by the Centre of Excellence on Partnership with Patients and the Public identifies patient roles at each of three stages in a learning cycle: patients may co‐develop data collection frameworks and tools, contextualise findings, and support the development, implementation, and communication of relevant solutions [17]. Patient involvement can also be supported by training to ensure that patients are equipped to contribute [14, 15] and that other interest‐holders within the system are equipped to create an “engagement‐capable environment.” [18] In an LHS, this training will need to address the broad array of activities that cross from data collection and analysis to implementation and evaluation.

The second distinguishing factor, namely exploration of evidence, is evident in papers describing the patient role in research within LHSs. However, in addition to research, evidence inputs within an LHS may also include analyses of administrative or practice‐based data sources such as electronic health records [3]. The engagement of patients with respect to these types of evidence and data has received much less attention in the LHS literature. Some authors have suggested that patients can play a role in raising awareness about data collection and patients' data‐related rights [7, 19]. However, one study found that patient advocates challenged the concept of “data” altogether, arguing that current routinely‐collected data does not reflect patient priorities [11]. As such, patients can play a role not only in collecting and analysing data, but also defining what types of data to collect—or even what constitutes “data” in a health context [11]. Given the centrality of the concept of data to LHSs, the role of patients in defining, collecting, and analysing administrative or practice‐based data within LHSs merits further elaboration.

Finally, the third distinguishing factor refers to an ecosystem of learning and supportive infrastructure. Organisations seeking to foster this learning‐intensive ecosystem will need to build capacity for patient involvement and avoid overburdening a small, active core of involved patients. While this poses a challenge, it also represents an opportunity. Some papers reference a core patient group that provided supports for involvement across an organisation [8], suggesting that a learning‐intensive environment can leverage or create opportunities for patient‐to‐patient collaboration, mentorship, and peer support.

### 1.2 Integration of Patient Knowledge

It is important not only that patients are present in LHS decision‐making structures and activities, but also that patients' knowledge meaningfully informs and changes those activities. For this to occur, there are relational, methodological, and epistemological concerns that must be addressed.

Relationships and their attendant power dynamics affect the extent to which patients have influence within LHSs. Some studies describe how patients actively contribute knowledge: for example, patients may be involved in prioritising topics for subsequent data collection [7], or identifying researchers' biases and contributing to nuanced analyses [9]. However, the inclusion of patients' knowledge cannot be assumed to follow inevitably from their presence [20]. Patients' priorities and values may conflict with those of other health system stakeholders [19], including those in positions of greater power. Moreover, conflict may arise not only between patients and other health system stakeholders, but also among patients themselves: as Irby and colleagues (2021) note, “working with one community does not yield one voice.” [10] To manage conflict, some papers suggest creating safe spaces for dialogue [8, 19]. More provocatively, Knowles and colleagues (2021) describe “constructive dissent” as a productive process that results in expanded conceptualizations [11]. These suggestions align with literature on participatory research and other domains of patient involvement [21, 22], where conflict has been found to be generative and essential. In fact, evading conflict can create tokenistic engagement where only those who agree with an established consensus feel free to speak [21]. Creating space for disagreement and dispute as well as consensus will demand skilful, creative, and attuned facilitation [23]. LHS practitioners need to attend to power dynamics and create space for disagreement to enable patients' involvement to result in new knowledge—and ultimately, new practices.

Methodologically, LHS practitioners must integrate patients' knowledge with research, clinical and administrative data. The question of how such integration can be accomplished is largely unaddressed in the current literature on patient roles in LHSs. Applying diverse knowledge types to a health system decision requires making sense of multiple inputs in relation to each other and to the particular LHS activity at hand. It has been argued that participatory research can adopt a pragmatic approach, where methods are chosen and combined in order to serve a particular, action‐oriented evidence need [24]; a similar argument can be made for LHS. However, adopting a naive “add and stir” approach to different worldviews can in fact re‐entrench traditional knowledge hierarchies that elevate positivism [25]. Health service organisations typically prioritise quantitative data over other information sources [20]. In the absence of guidance for navigating diverse ways of knowing and value systems, LHSs may default to familiar biomedical and/or positivist approaches, rendering patient involvement tokenistic or even exploitative. Indeed, LHS literature has been critiqued for treating patients as “data donors” or passive sources from which information can be extracted [4]. For instance, some LHS literature describe collection and use of patient‐reported outcome and experience measures as an enabler of patient engagement [26]. However, the implementation of these measures—in the absence of meaningful partnership with patients throughout the learning process—does not in fact reflect the integration of patient knowledge. Rather, it aligns with traditional approaches in which patients are treated as subjects about which organizational or biomedical knowledge may be generated.

Counteracting this risk will involve grappling with underpinning epistemological complexity. Shifting epistemological hierarchies requires acknowledgement of the diverse knowledge systems at play. This argument has been made in the context of interdisciplinary teams [25], but applies equally to LHSs. Beyond acknowledgement of diverse epistemologies, integrating patient knowledge demands attention to the question of what patient knowledge entails. In one study, authors suggest complementing “big data” with rich patient narratives. These narratives can provide a check on the meaning of more traditional types of evidence, but also serve as an important type of knowledge in their own right [11]. Patient knowledge, however, eludes simple definitions. There is no singular experience of “being a patient” [27] or of having a particular diagnosis [28]. Patient knowledge instead arises from patients' changed relationship to social and material contexts [28], a shift often defined by vulnerability [27]. Moreover knowledge is not an automatic byproduct of experiences of marginalisation, but rather is developed through critical reflection [29]. Patient knowledge has been described as both embodied [27, 30] and collective [28] or relational [30]. The ongoing definitional debates and complexity surrounding patient knowledge must be surfaced and explored to inform LHS methodologies that meaningfully transform multiple types of knowledge into action.

### 1.3 Recommendations for Research and Practice

It is well established that patients should be central to LHSs. However, this centrality is not always realized in practice, or clearly defined [7, 20]. Firmly embedding patient involvement in LHSs will require appropriate structures and roles, along with clear practices for integrating patient knowledge in decision‐making and data analysis. It also requires commitment, capacity, and skills within the health system [4]. Future research, including theoretical development and detailed case studies, can add to our knowledge on the breadth and depth of patient roles within LHSs, and the strategies used to ensure that patient knowledge is woven into health system learning. In keeping with the ethos of LHSs, this academic work must be interwoven and concurrent with the actual practice of health system learning.

Based on the exploration above, we make six recommendations for integrating patients and patient knowledge into LHSs.

1. Patient involvement in LHSs should be supported by co‐constructed training that accounts for the full array of activities across a learning cycle.

Training of patients and other LHS practitioners can support patients' confidence and facilitate their full involvement in health system activities. LHSs span a broad range of activities, and patient partners in LHSs will therefore have broad knowledge needs. This is also true of other members of LHS teams, such as clinical staff. Training and onboarding should prepare patients to engage with all stages of LHS work from data collection and analysis to implementation, evaluation, and iteration of innovations; it should also prepare other LHS practitioners to engage effectively with patients through this process. Moreover, as noted above, the initial development of the LHS concept lacked patient involvement [4]. It cannot be assumed that patients will be passive recipients of this training or the concepts that underpin it, but rather may offer critical insights into weaknesses and gaps [11]. As such, training should be co‐constructed with patients and should allow for the framing of an LHS and attendant learning needs to shift as a result of patient input. Ontario SPOR Support Unit offers co‐developed trainings on Learning Health System concepts aimed at organizational, policy, and lived experience leaders [31]. Meanwhile, a wide range of co‐developed training resources focused on research (e.g. the Patient Experience Research Centre's Peer Research Training Resource) [32], quality improvement (e.g. SSA Quebec's online training on patient engagement in continuous quality improvement) [33], or related activities can be bundled, adapted, and iterated to address learning needs within LHSs.

2. Organisations should build capacity for patient involvement across the broader LHS environment.

A defining feature of LHSs is a learning‐intensive environment, in which multiple learning cycles operate concurrently [16]. If patients are to be key players in all learning processes, organisations will need to build capacity to make this possible. Capacity‐building efforts should be led by, or at minimum carried out in partnership with, existing patient leaders. A learning‐intensive environment also creates opportunities for patients to work together and support each other across LHS activities. Structures and processes for patient partner collaboration and peer support should be built as part of LHS implementation. Finally, efforts to build capacity should be attentive to diversity and power dynamics within health systems, with an aim of enabling the involvement and leadership of patients with intersecting marginalised identities.

3. Approaches for engaging patients in defining, collecting, and analysing health data should be developed and disseminated.

To date, there is minimal literature that documents the role of patients in defining, collecting, and analysing administrative and routinely‐collected health data in an LHS—despite the centrality of data in LHS discourse and practice. A few possible roles have been described: for instance, patients may offer valuable insight into types of data that matter most [11, 19], or support the development of strategies that enable other patients to understand their rights relating to information collection [7, 19]. Meanwhile outside of LHS literature, there is an emerging focus on patient and public involvement within “big data” initiatives that may have relevance for LHSs. For instance, Nelson and Burns (2020) describe the involvement of voluntary sector organisations representing populations implicated by the work of the Administrative Data Research Centre Northern Ireland; they identify this as a first step to building relationships and trust to enable involvement of directly‐affected members of the public [34]. Case studies offering rich descriptions of patient involvement in data‐based LHS functions will be a valuable contribution to the field.

4. LHSs should create space for productive disagreement as well as consensus.

LHSs involve collaboration among diverse stakeholders with diverse worldviews. Diversity is a boon—but only if participants are able to bring their viewpoints forward. As such, LHSs must establish spaces for dialogue in which dissent is embraced, even as consensus is sought. Building such a space requires appropriate facilitation [23]. It also requires humility on the part of health system stakeholders [23]: those participating in an LHS must be willing to recognise, name, and disrupt power dynamics that can suppress dialogue. As noted above, participatory research traditions engage richly with the value of dissensus. Productively surfacing and channelling dissent within LHSs—where it will have implications not only for interpretation of findings, but for the subsequent translation of findings into action—merits further exploration and representation in the literature.

5. Making sense of patient knowledge within LHSs, in itself and in relation to other ways of knowing, will require grappling with complexity.

Treating patient knowledge as a mere addendum to research and health system data will result in further marginalisation. Meaningfully integrating diverse forms of knowledge, however, is a complex task. Integrating patient knowledge ought to change the way an issue is understood within an LHS—not simply by providing additional information, but by changing the meaning of other forms of evidence within a dynamic interpretive process. As with dissensus, academic literature on participatory research offers rich resources for conceptualising the meaning of patient knowledge. However, worked examples of how these conceptualizations play out in real‐world, real‐time decision‐making are limited. Theoretical exploration can be undertaken to elaborate the epistemological dimensions of patient involvement in LHSs. This can in turn inform methodological development to guide the integration of diverse ways of knowing within learning health systems; and case studies of how these concepts and methodologies are applied and refined.

6. Researchers, practitioners, and patient partners should co‐develop measures for evaluating patient involvement and leadership in LHSs

In a high‐functioning LHS, practice change is guided by the use of real‐time evidence, including the experiential knowledge of patients. To ensure the centrality of patient knowledge, teams should develop shared process and outcome measures of patient involvement and leadership in LHSs. For example, these measures could explore patient perspectives of meaningful engagement, degrees and perspectives of meaningful patient involvement from all LHS stakeholders, and longer‐term outcomes related to how, or if, patient perspectives helped to inform or change healthcare practices. Existing evaluation resources focused on involvement more broadly such as the Patient and Public Engagement Evaluation Tool [35] or the Co‐Production Reflective Resource Tool [36] may be adapted to address unique aspects of LHSs. Evidence on successful patient engagement should be woven into learning cycles to guide genuine and lasting patient involvement and leadership.

## Ethics Statement

The authors have nothing to report.

## Conflicts of Interest

The authors declare no conflicts of interest.

## Data Availability

Data sharing is not applicable to this article as no new data were created or analyzed in this study.
